# Book Reviews

**Published:** 1876-05

**Authors:** 


### Book Reviews.

[NOTE.—All works reviewed in the pages of the CHICAGO MEDICAL JOURNAL AND EXAMINER may be found in the extensive stock of W. B. KEEN, COOKE & Co., whose catalogue of Medical Books will be sent to any address upon request.]

CYCLOPÆDIA OF THE PRACTICE OF MEDICINE. Edited by *Dr. H. Von Ziemssen*, Professor of Clinical Medicine in Munich, Bavaria. Vol. III. Chronic Infectious Diseases, by *Prof. Christian Bäumler*, of Erlangen; *Prof. Arnold Heller*, of Kiel; and *Prof. Otto Bollinger*, of Munich. Translated by Arthur D. Nichols, M.D., of Boston, Wm. Ashbridge, M.D., of Philadelphia, James G. Hyndman, M.D., of Cincinnati, and Edward B. Bronson, M.D., and Edward L. Keyes, M.D., of New York. Albert H. Buck, M.D., of New York, Editor of American Edition; pp. 672. Wm. Wood & Co. New York. 1875.

LECTURES ON SYPHILIS AND ON SOME FORMS OF LOCAL DISEASE AFFECTING PRINCIPALLY THE ORGANS OF GENERATION. By *Henry Lee*, Professor of Surgery at the Royal College of Surgeons, England, etc. Philadelphia: Henry C. Lea. 1875. Pp. 246.

In this our day, when the literature of every medical subject is rapidly multiplying, there are only two species of books valued highly by the professional reader. One class embraces the cyclopædic works—literally understood. They attempt to traverse the cycle of knowledge, attained or attainable, on the subject of which they treat. An imperfect accomplishment of the orbit detracts from their value, in proportion as the failure occurs at those points where it is particularly necessary that the object in view should be attained.

In a second class of books, less pretentious, the author is absolved from the necessity of carefully collecting the treasures which have been mined by others before him, because of the genuine value of his own contribution. It must be original, either in matter or in mode of presentation, in order to secure success.

The volumes named above belong each to one of these classes, and judged from the standard suggested, each must be decided to be deficient in the elements which give permanent value to any work.

Over three hundred pages of the Cyclopædia are allotted to Professor Bäumler's treatise on syphilis—space which should suffice for a condensed review of the literature of the subject. And yet, on many topics which require the most careful elucidation with a comparison of authorities, the details given are meagre in the extreme. Where the author attempts to submit original observations, we do not think the value of the treatise has been greatly enhanced.

Thus, for example, in considering the primary affection in syphilis, twenty-three pages are devoted to the discussion of the doctrines of unity and duality—a discussion in which the ideas of the two schools are very fairly presented—while but four pages are allotted to the description of the primitive lesion itself. Of these, one and one-half pages are taken up in describing the flat papule of inoculation, before the attention is directed to lesions of mucous surfaces. Here the author takes a position in which he stands nearly if not quite alone. He describes "a small, itchy vesicle upon a reddened base, or an erosion, due to the bursting of the vesicle, which could not be distinguished from the erosion resulting from a simple herpetic vesicle, such as often occurs upon the inner layer of the prepuce," as the first thing observed after the period of incubation. Dubuc attracted considerable attention in 1874, when his article on "multiple herpetiform chancres" first appeared in the *Annales de Dermatologie et de Syphiligraphie*, but he did not then claim, nor do we know that others since his writing have claimed, that the vesicle was generally the initial stage of the initial lesion of syphilis. Neither, we think, can it be demonstrated that the superficial erosion, which is the insignificant and most common form of the earliest manifestation of the disease, results from the rupture of a minute vesicle. The smooth, polished, and often pigmented surface, of the color of raw ham (Swediaur) which is the first phase of many chancres, does not in any way suggest the little ulcer to be found at the base of a "simple herpetic vesicle." Minute as it is, and apparently insignificant as it is, it is fraught with intense interest to the student of the career of syphilis, and deserves a much more careful description than that accorded to the entire subject, in the volume under consideration. The various modifications of the primary sore, according as it occurs upon the os uteri in woman—so carefully delineated by Fournier—or as there is developed upon and from it the well marked forms of the ulcerating chancre, are not discussed.

The primary lesion, in several parts of the treatise, is denominated the "syphilitic initial sclerosis." There is, no doubt, sufficient confusion in the nomenclature of venereal ulcers to justify the introduction of a new name by which they can be more clearly identified, but that proposed by Bäumler wants the exactness of a definitive term. "Sclerosis" is a common but not essential feature of a primary syphilitic sore. It is in fact an accident in its development. "A chancre," says Fournier, "is indurated because it is syphilitic, not syphilitic because it is indurated." Induration is true of nineteen out of twenty infecting ulcers, but the twentieth in which it is lacking, demonstrates the fact of this misnomer.

We have referred to the deficiencies of the treatise in this particular instance, merely to indicate one of many subjects whose treatment is eminently unsatisfactory. The subjects of the syphilides, their diagnostic features and treatment, syphilis of the viscera and nervous system, and hereditary syphilis, are loosely handled and imperfectly developed. Nothing, for example, could be more disappointing to a practitioner confronted with his first case of syphilitic iritis, than to consult the single page which Prof. Bäumler has written under this title, and upon which not one word appears which gives the slightest clue to the treatment which of late years has proved so conspicuously satisfactory, even when distinct syphilomata have been found upon the pupillary margin.

The latter part of the volume, which treats of infection by mineral poisons, and the diseases from migratory parasites, is well illustrated and supplies a valuable contribution to the literature of a subject which is yet, to a great extent, obscure.

The translation of the whole work has been admirably accomplished, and we can not but wish that one of the translators, at least, who has made for himself an enviable reputation as an author in syphilography, had added some chapters of his own writing. There are some typographical errors which a more careful proof-reading should have corrected, but in the general excellence of the work of the publishers, this volume is quite equal to any of its predecessors.

The Lectures on Syphilis belong to the second of the two classes of books we have described, and the key note of the work is sounded in the very first sentence of the preface: "The principal object of the present work is to illustrate some of Hunter's doctrines which the lapse of time and the dissemination of more recent views have obscured, or caused to be forgotten." This may be a praiseworthy effort, but we think it misdirected, and one not at all promising for the reputation of the author. If Mr. Henry Lee had set himself to study syphilis for its own sake, and with the single view of teaching himself and his fellows the truth respecting it, he would have accomplished vastly more good. As it is, he has looked at chancres through an Hunterian objective—never an achromatic lens, and comparing unfavorably with its rivals of to-day. The profession, the scientific world and the world at large, will ever hold in the highest reverence the name of John Hunter, but they realize the fact that the medical fledgling of 1875 knew more of the truth about syphilis than ever did John Hunter.

Mr. Henry Lee at one time attracted considerable attention by his experimental inoculations with the secretions of inflamed chancres, but we do not find that since then his efforts have contributed in any direction to the advancement of syphilology. The distortion of mental vision produced by his effort to illustrate the doctrines of Hunter has rendered the lectures on syphilis obscure and valueless to the student. The inferences drawn from the cases cited are partly conjectural and partly due to misinterpretation of clinical facts.

"The principal constitutional cause," he says, "which modifies the first appearance of a real syphilitic inoculation, is the existence of what I have, for want of a better term, called the syphilitic fever." We do not know, when we read these lines, whether to account for their apparent disingenuousness by supposing the writer to be exceptionally audacious or ignorant. But the doubt is removed on the very same page (52), where we read further, that the inoculation of common pus will, under ordinary circumstances, not produce a pustule; while "under the influence of this fever" it may give rise to it. Long before these words were penned, the doctrine of the contagiousness of all common pus, with or without fever, had been promulgated by Van Roosbroeck and demonstrated by Pick, Chauveau and others. The professor of surgery at the Royal College of Surgeons, ignorant of these facts or ignoring them (the culpability being in either case the same) has thus produced a work in which an essential error has marred almost every explanation, detail and didactic statement. It is full of crudities, and misconceptions—all resulting from a single source—the egregious folly of looking at nature in health and disease not in order to observe the truth, but to obtain some little modicum of verity which will substantiate a preconceived opinion.

We know of no value attaching to these lectures beyond the addition they furnish to the clinical material of syphilitic cases.

Extra-Uterine Pregnancy; its Causes, Species, Pathological Anatomy, Clinical History, Diagnosis, Prognosis, and Treatment. By *John S. Parry, M.D.*, Obstetrician to the Philadelphia Hospital, etc., etc. 276 pages. Philadelphia: Henry C. Lea. 1876.

This very excellent book is based upon an analysis of five hundred cases collected from various sources, and, for a work of its size, shows immense research, and, as far as we can judge, perfect fairness in giving credit to the multitude of authors to which it refers. After confessing that in many cases the cause of this distressing difficulty cannot be determined, the author places *previous pelvic disease* at the head of the list, as being the probable cause in many instances. Inaptitude to conception, hernia of some portion of the internal genital organs, ordinary uterine displacements, and tumors, are also mentioned as causes. The particulars of two remarkable cases are given, where, after surgical operations, openings having been made through the uteri, the fertilized ova escaped into the abdominal cavity, and pregnancy took place.

Our author objects to the "minute anatomico-pathological" classification of Dezeimeris and others, and produces one of his own. He believes that there are three species of extra-uterine pregnancy, viz., tubal, ovarian, and ventral or abdominal; each species being divided into several varieties. The three recorded cases of supposed vaginal pregnancies are carefully reviewed, and the paragraph closes with the following sentence:

"It may, therefore, be concluded that we have no reliable clinical evidence that vaginal pregnancy is possible, and that there are good reasons for believing that it can not occur."—p. 49.

The *pathological changes* (1) during the early stage, (2) during the later months of gestation, and (3) after its close, are carefully considered, occupying about forty pages of the book. It seems to be well established that in these erratic pregnancies, the uterus undergoes changes nearly similar to those taking place when the pregnancy is normal. In regard to the formation and discharge of the decidua, three propositions are given, which may be epitomized as follows: "A decidua is always formed in extra-uterine pregnancy in the uterine cavity. It is rarely retained until completed gestation, being more frequently thrown off during the early months, with symptoms of miscarriage. Its absence, when death takes place from rupture of the cyst, is no proof that it has not been formed—it has probably been previously expelled."—p. 72.

The *symptoms* of extra-uterine pregnancy are studied under three divisions, the author giving particular importance to the signs of the first period. This stage includes the first months of pregnancy, previous to the time when the sounds of the fœtal heart can be detected, and is characterized by the patient supposing herself to be pregnant, and by hypogastric, colicky pains, often so extremely violent as to produce syncope, with menorrhagia.

The symptoms during the second period, and the phenomena present after the end of the normal time of gestation, are carefully considered.

The various theories as to the cause of pains, somewhat like if not identical with those of labor at the end of normal pregnancy, are also discussed. Against the theory of Powers, proposed in 1819, that of Brown-Sequard, in 1855, of King, of Washington, D.C., in 1871, and of Clarke, of Oswego, N. Y., in 1872, our author sees "insuperable objections," and rejects them all. The cause of labor pains at the close of extra-uterine gestation is not accurately known. The theories have been numerous. There is, however, now some clinical evidence (more being necessary to establish it as absolutely certain), which makes it probable that these pains are due to contractions of the uterus.

The *termination* of an extra-uterine pregnancy, we are assured by the author, is not without interest, "whether viewed with regard to the marvellous circumstances under which life is sometimes preserved, or the frightful rapidity with which it is destroyed in other cases." The subject is considered under the following heads: (1) Rupture of the Cyst; (2) Changes which follow Retention of the Fœtus for a Long Period; and (3) Discharge of the Child through the Bowel, Bladder, Vagina, or Abdominal Wall.

The *mortality* is frightful. In the 500 recorded cases 336 died—a death rate of 67.20 per cent.

The *differential diagnosis* is considered at length, and, in view of the fact that many of our most eminent obstetricians have been misled in their examination of these cases, it is a subject that should receive our most diligent study. Extra-uterine pregnancy is liable to occur in the practice of every medical gentleman, and the careful study of 500 recorded cases by any one cannot fail to be of great service.

The consideration of *the treatment* occupies seventy-five pages of the volume, and, in addition to palliative measures, the comparative value of the following methods, during the first period, are discussed: 1, Destruction of the ovum through the system of the mother; 2, Extirpation of the fœtal sac; 3, Puncture of the fœtal cyst; 4, Removal of the embryo by section of the vagina with the galvanic cautery; 5, Galvanism and electricity; 5, The injection of narcotic substances into the cyst; 7, Compression of the tumor.

The last pages of the book are devoted to a consideration of the operation of gastrotomy after rupture of the cyst, which the author not only believes justifiable, but says it is "simply criminal to sit idly by and see a woman die from rupture of an extra-uterine fœtal cyst without attempting to save her."

In a careful perusal of the volume we have noticed but a single typographical error.

In conclusion, we must say that we are proud of the book. It is a valuable addition to American medical literature. We honor the man who has so patiently wrought out from such a vast amount of material so valuable a work, which should be in the possession of and read by every practitioner. C. W. E.

---

A Synopsis of the Symptoms of Gout at the Heart. Also, a few Practical Remarks on Epilepsy, Nervousness, and other Kindred Diseases in relation thereto. By *Eldridge Spratt.*

We have just finished a perusal of this book, and are justly amazed at its contents. At first we were inclined to believe it written by some clever fellow as a satire upon medicine, something after the manner of Le Sage, Molière *et al.;* but a more careful study, and the fact of its having reached twelve editions, lead us into a more serious train of thought, and incline us to give it a more thorough inspection. It seems to us hardly a credible fact that, at this age of medicine, and with such advances as have been made in pathology, physiology, and kindred matters, that a physician could be found—and in England too—to produce such a trashy work, with such a lack of true medical knowledge. It speaks badly for the professional *status* of its readers, and we can only hope that his confrères (as he terms them), Sieveking, Tanner, Garrod, Fuller, the late Drs. Skey, Todd, Wardrop and others, were physicians of too kind hearts, and too indulgent to wound his sensibilities by advising its suppression, before the issue of its second edition.

There is not an original idea in the whole, and it is a work of surprises and disappointments, being only relieved by the extracts from good writers, and the anatomical plate in the beginning. We do not understand how so respectable a firm as J. B. Lippincott & Co. could have been persuaded into publishing such a muddy and weak affair, giving, as it were, a *quasi* endorsement of its demerits, and for which many a professional man will not be at all thankful. F. B. N.

BOOKS RECEIVED.

CYCLOPÆDIA OF THE PRACTICE OF MEDICINE. Edited by *Von Ziemssen.* Vol. IV—Diseases of the Respiratory Organs.

DISEASES OF THE NOSE AND ITS ACCESSORY CAVITIES. By *W. Spencer Watson, F.R.C.S., Eng.*, B. M. London.

SAINT BARTHOLOMEW'S HOSPITAL REPORTS. Vol. XI. Edited by *James Andrews, M.D.*, and *Thomas Smith, F.R.C.S.*

SURGERY OF THE ARTERIES. By *C. F. Maunder*, Surgeon, etc.

THE STUDENT'S GUIDE TO THE PRACTICE OF MIDWIFERY. By *D. Lloyd Roberts, M.D., M.R.C.P.* London.

A TREATISE ON THE DISEASES OF THE NERVOUS SYSTEM. By *William A. Hammond, M.D., etc.* Sixth edition.

MORTUARY EXPERIENCE OF THE MUTUAL LIFE INS. CO., of New York. From 1843 to 1874.

A MANUAL OF DISEASES OF THE EYE. By *C. Macnamara, F.C.M., etc.*

CLINICAL LECTURES ON DISEASES OF THE HEART AND AORTA. By *Geo. Wm. Balfour, M.D., St. And., F.R.C.P., Ed., etc.*

PAMPHLETS RECEIVED.

SPECIMEN FASCICULUS OF A CATALOGUE OF THE NATIONAL MEDICAL LIBRARY. Compiled by *Dr. J. S. Billings*, Asst. Surgeon, U. S. A.

HISTORICAL SKETCH OF UNION COLLEGE, Washington: Government Printing Office. 1876.

